# CRISPR-Cas9-mediated editing of *ZmPL1* gene improves tolerance to drought stress in maize

**DOI:** 10.1080/21645698.2024.2448869

**Published:** 2025-01-17

**Authors:** Chunlai Wang, Yangyang Zhou, Yimeng Wang, Peng Jiao, Siyan Liu, Shuyang Guan, Yiyong Ma

**Affiliations:** a College of Agronomy, Jilin Agricultural University, Changchun, China; b Joint Laboratory of International Cooperation in Modem Agricultural Technology of Ministry of Education, Jilin Agricultural University, Changchun, China

**Keywords:** CRISPR-Cas9, drought, maize, Phylloplanin-like (*ZmPL1*)

## Abstract

Maize (*Zea mays* L.) is a widely grown food crop around the world. Drought stress seriously affects the growth and development process of plants and causes serious damage to maize yield. In the early stage, our research group conducted transcriptome sequencing analysis on the drought-resistant maize inbred line H8186 and screened out a gene with significantly down-regulated expression, Phylloplanin-like (*ZmPL1*). The *ZmPL1* gee expression pattern was analyzed under various abiotic stresses, and the results showed that this gene was greatly affected by drought stress. Subcellular localization analysis showed that the protein was localized on the cell membrane. In order to verify the role of *ZmPL1* in drought stress, we overexpressed *ZmPL1* in yeast and found that the expression of *ZmPL1* could significantly increase the drought sensitivity of yeast. Next, *ZmPL1* transgenic plants were obtained by infecting maize callus using Agrobacterium-mediated method. Under drought stress, compared with overexpression lines, gene-edited lines had higher germination rate and seedling survival rate, lower accumulation of MDA, relative conductivity and ROS, higher antioxidant enzyme activity, and the expression levels of stress-related genes and ROS scavenging-related genes were significantly increased. Exogenous application of ABA to each lines under drought stress attenuated the damage caused by drought stress on *ZmPL* overexpressing plants. In summary, *ZmPL1* negatively regulates drought tolerance in maize.

## 1. Introduction

Maize is an important food, cash and energy crop in the world and plays a very important economic role. In recent years, the bad climate has greatly increased the frequency of drought, seriously affected the normal growth and development of corn, and restricted the yield of corn.^1^ Therefore, it is of great significance to mine maize drought tolerance genes to improve maize drought tolerance and increase maize yield.

Under drought stress, roots are the first to detect water shortage signals. Root hairs enhance the root surface area, thereby improving plant water uptake efficiency.^2,3^ In *Populus hopeiensis*, the *SpsNAC042* gene promoted root development and enhanced the tolerance of *P. hopeiensis* to stress.^4^ At the same time, the leaf shape will also undergo dramatic changes, which is generally manifested as curling and yellowing of the leaves. During this process, plants reduce water loss by closing stomata.^5^ As drought stress intensifies, the activity of enzymes involved in photosynthesis diminishes, and the photosystem is disrupted, leading to the accumulation of reactive oxygen species (ROS) within the plant, thereby causing oxidative stress.^6,7^ Plants have formed some antioxidant systems in the long-term evolution. SOD, CAT, etc., are essential antioxidant enzymes when plants encounter drought stress, which can reduce the damage of ROS to plants.^8^ In *Prunus mira Koehne*, *PmNACA1* played an active role in drought stress response by scavenging ROS.^9^ In *Arabidopsis*, the miR408-PCY module regulated ROS homeostasis and drought tolerance by transmitting ABA signaling.^10^ Malondialdehyde (MDA) is one of the products of membrane peroxidation. High levels of MDA can damage the cell membrane structure.^11^ In apple, overexpression of *MdNAC29* reduced drought tolerance in apple, callus, and tobacco plants, showing higher relative conductivity, MDA content, and lower chlorophyll content under drought stress.^12^ In tomato, overexpression of the tomato *SlUGT73C1* gene in *Arabidopsis* increased the seed germination rate, increased SOD and POD activities, decreased MDA content, and increased the expression levels of osmotic stress regulatory genes and rate-limiting enzyme genes in the proline biosynthesis pathway.^13^ Drought stress induces responses in endogenous hormone signaling pathways in plants, with abscisic acid (ABA) serving as a pivotal regulator of plant growth, development, and adaptation to drought conditions.^14^ Exogenous application of ABA has been shown to enhance ABA synthesis in plants under drought stress, thereby regulating stomatal closure, minimizing water loss, and improving plant resistance and defense mechanisms against drought stress.^15^ For example, the overexpression of *MdABI5* enhanced ABA sensitivity and drought tolerance by directly activating the expression of *MDM6* and *MdRD29A*. *MdTCP46* significantly inhibited the transcriptional activity of *MdABI5*, thereby negatively regulating *MdABI5*-mediated ABA signaling and drought response.^16^ In rice, the inactivation of the transcription factor *OsWRKY5* enhanced drought tolerance through ABA signaling pathways.^15^

The Phylloplanin-like gene encodes a secreted tolerance protein with a unique transmembrane signal peptide structure, playing a crucial role in regulating plant responses to biotic and abiotic stresses.^17^ The function of the Phylloplanin (PL) gene has been reported to rely on the sulfur and glutathione (GSH) defense mechanisms in response to oxidative stress.^18^ Recent studies revealed that the Phylloplanin gene in tobacco synthesizes a unique protein known as T-Phylloplanin-like protein.^19^ Phylloplanin-like proteins have been demonstrated to enhance plant resilience against abiotic stresses, including drought, salinity, and cold stress.^20^ Antoaneta B et al.^19^ used RNAi technology to study the function of the Phylloplanin gene, mainly analyzing the glycoprotein characteristics and renewability of the Phylloplanin protein. Meanwhile, the Phylloplanin protein in sunflower and Setaria contained the same specialized protease activity as that in tobacco, enabling them to respond effectively to abiotic stress. Choi et al.^21^ found that Phylloplanin protein exhibited highly glycosylated and hydrophobic properties and identified several protein sequences with high homology to Phylloplanin protein in the EST library. Phylloplanin genes may be involved in the process of plant response to abiotic stress, but the specific biological function is still unclear. Wang et al.^22^ found that tobacco varieties TI1112 and TI1406 secreted a large amount of phylloplanin genes when they lacked TGSTs. In summary, the biological structure and function of phylloplanin genes in plants, particularly in maize, remain largely unknown.

In recent years, CRISPR gene editing technology has advanced rapidly and has been widely applied to gene modification and editing across various species, establishing itself as a key tool in reverse genetics research.^23^ The CRISPR/Cas9 gene editing technology, derived from the type II CRISPR/Cas system in prokaryotes, offers several advantages, including low development costs, ease of construction, and high editing efficiency.^24^ For example, CRISPR-Cas9-mediated editing of *GmARM* improved tolerance to multiple stresses in soybean.^25^ The CRISPR/Cas9 technology was employed to knock out the *ZmATHB-6* gene while simultaneously overexpressing it. The study revealed that overexpression of *ZmATHB-6* improved maize tolerance to drought.^26^ In this study, CRISPR/Cas9 technology was utilized to analyze gene function.

In this study, significantly down-regulated differential gene *ZmPL1* was obtained from the transcriptome database of the laboratory prior drought. The *ZmPL1* gene was transformed into maize plants using the Agrobacterium-mediated transformation method. The function of the *ZmPL1* gene under drought stress was analyzed. The results showed that, compared with the overexpression lines, the gene-edited lines had a higher germination rate and seedling survival rate, lower accumulation of MDA, relative conductivity and ROS, higher antioxidant enzyme activity, and significantly increased expression levels of stress-related genes and ROS scavenging-related genes. Therefore, *ZmPL1* gene editing can significantly improve drought tolerance of maize.

## 2. Materials and Methods

### 2.1. Bioinformatics Analysis

The sequence information of *ZmPL1* gene was obtained from the public database NCBI. The whole-genome data of maize sequences were downloaded from maizeGDB. The whole-genome data of *Triticum aestivum*, *Setaria italica*, *Sorghum bicolor*, *Oryza sativa*, and *Arabidopsis thaliana* sequences were downloaded from Ensembl. The phylogenetic analysis of ZmPL1 and homologous genes from other species was performed by aligning the sequences using default parameters in CLUSTAL W. Phylogenetic tree was constructed using MEGA version 7 with neighbor-joining. The constructed phylogenetic tree was visualized using iTOL version 6. Cis-acting elements within the 2000 bp upstream promoter sequence of *ZmPL1* were identified and analyzed using PlantCARE.

### 2.2. Growing Conditions of Plant Material and Stress Treatment

The maize inbred line H8186 and maize callus GSH9901 used in the experiment were provided by the Biological Joint Laboratory of Jilin Agricultural University. Using maize H8186 as the material, 48 seeds were taken and three seeds were sown in the same plastic pot (20 cm × 25 cm), for a total of 16 pots.The plants were watered and sprayed with nutrient solution once per week and cultivated in a greenhouse under controlled conditions of 25 °C/16°C (day/night temperatures), a 14 h/8 h light/dark cycle, and 65% relative humidity.

For the drought stress treatment, when the maize plants reached the three-leaf stage, the experimental group was subjected to natural drought conditions. Specifically, watering was stopped when the soil relative humidity reached 70% and continued until it dropped to approximately 30%, representing a state of severe drought. In contrast, the control group was maintained at a consistent soil relative humidity of 70%. Plant survival was assessed by observing the leaves; plants were considered alive if the majority of their leaves remained green and had not completely dried out.

For seed germination assays under drought treatment, maize seeds from each lines were placed individually on filter paper in Petri dishes. The Petri dishes were divided into two groups, each containing wild-type (WT), overexpression, and knockout seeds. Sterile water was added to one group as the control, while a 15% PEG6000 solution was used for the treatment group. Germination was counted after 5 days of germination. In addition, the root lengths of each lines were measured. Seed Germination Criteria: Germination was defined as successful when the root length exceeded the full length of the seed or when the shoot length surpassed half the length of the seed.

For ABA treatment, the seedling experiment was carried out when the maize grew to the three leaf stage, and 100 μM ABA was sprayed on the leaves daily during drought treatment. Phenotypes were observed at 14 days after treatment. For germination tests, the air-dried seeds of both WT and transgenic wheat plants were sown in Petri dishes containing distilled water with or without ABA treatment. Germination percentages were determined after a 7 days incubation at 22°C.

### 2.3. *Analysis of* ZmPl1 *Gene Expression Pattern*

The expression of the ZmPL1 gene in various maize tissues (root, stem, and leaf) was analyzed through sample collection and detection. The plants were placed in a 4°C incubator for low temperature stress, placed in a 15% PEG6000 solution for drought stress, and placed in a 150 mm/L NaCl solution for salt stress. The leaves of the plants were sampled at 0 h, 2 h, 4 h, 6 h and 8 h after the above stresses, quickly frozen with liquid nitrogen, and stored in a −80°C refrigerator. Plant RNA was extracted using the Trizol method and cDNA was synthesized using a reverse transcription kit. cDNA was used as the template, and *ZmACTIN1* was used as the internal reference gene. The PCR reaction system was 25 μL consisting of 2 μL cDNA, 1 μL forward primer, 1 μL reverse primer, 12.5 μL qRT-PCR Master Mix, and 8.5 μL sterilized ddH_2_O. The reaction procedure was as follows: 95°C, 10 min, 1 cycle; 95°C 10 s, 60°C 20 s, 72°C 15 s, 40 cycles; 95°C 1 min, 55°C 30 s, 95°C 30 s.^26^ Gene expression was calculated by 2^−ΔΔCt^ method. Each sample was repeated three times.

### 2.4. Generation of Constructs and Transgenic Plants

The pCAMBIA3301-ZmPL1 overexpression vector was constructed with restriction sites of BstE II and Bgl II. The pCXB053-ZmPL1 vector was constructed by Weimi Biotechnology Co., LTD. (Jiangsu) with the target sequence of CCCTCGTGCAGCTCGTGTGCGG. The above two recombinant plasmids were transformed into maize callus GSH9901 by *Agrobacterium* infection. The positive plants were screened using PCR technology, and the target site mutations in the gene-edited lines were analyzed. (Supplementary Figure S2, S3). Through the above analysis, three independent lines with *ZmPL1* overexpression and three independent lines with gene-edited *ZmPL1* were screened(Supplementary Figure S4). The primers used in this study are listed in Supplementary Table S1.

### 2.5. Subcellular Localization

The pCAMBIA1302-ZmPL1 vector (containing GFP) was constructed with restriction sites Bgl II and Nco I. After the successful construction, the plasmid was transformed into *Agrobacterium tumefaciens* GV3101 competent cells, and subsequently transiently transformed into the young leaves of *Nicotiana benthamiana*. The localization of the protein was observed using a confocal microscope.^27^ The primers used in this study are listed in Supplementary Table S1.

### 2.6. *Functional Verification in* Saccharomyces cerevisiae *Cells*

The vector pYES2-ZmPL1 was constructed by homologous recombination, and the successfully constructed vector and the empty vector pYES2 were respectively transferred into the *Saccharomyces cerevisiae* INVSc1. The transformation products were coated on SD/-Ura plates and incubated at 29°C for 2–3 days to screen for positive clones. The yeasts carrying different plasmids were diluted at different concentrations and piped up 10 uL onto SD/-Ura and SD/-Ura +1.2 M Mannitol, respectively.^28^ The primers used in this study are listed in Supplementary Table . S1

### 2.7. Physiological and Biochemical Indexes of Transgenic and WT Lines Were Detected Under Drought Stress

The contents of H_2_O_2_ and O_2_^−^ were determined as previously described.^29,30^ The tip of a maize leaf was taken and immediately placed in a centrifuge tube containing NBT dye solution. It was left to stand at room temperature for 60 min, after which the NBT dye solution was discarded. 95% ethanol was added and decolorized in a water bath at 80°C. 95% ethanol was changed every 10 min. After the green color of the sample completely faded, the sample was removed, and the staining results were recorded. DAB detection method: The isolated leaves were immersed in DAB dye solution, incubated at room temperature overnight, decolorized with 95% ethanol in 80°C water bath until clarified, and photographed for observation. The chlorophyll content detection method was referred to the chlorophyll content detection kit. The malondialdehyde (MDA) content detection method was referred to the MDA content detection kit; the superoxide dismutase (SOD) activity detection method was referred to the SOD activity detection kit; the peroxidase (POD) activity detection method was referred to the POD activity detection kit. Relative conductivity (REC) : Detached leaves were collected and washed three times with deionized water followed by shaking in deionized water for 2 h. Then the conductivity was measured as initial data (S1), and then samples were boiled for 10 min and conductivity was measured again as final data (S2) when the solution cooled down to room temperature. The relative electrical conductivity (REC) was calculated as follows: REC (%) = S1/S2 × 100.^31^ Relative water content: The fresh weight of the leaves was recorded as m1. Then, the leaves were placed in a 50 mL centrifuge tube, soaked in ddH_2_O for 2 h. Afterward, the surface water of the leaves was wiped dry, and the leaves were weighed and recorded as m2. Finally, the leaves were dried in a drying oven at 60°C, and the dry weight was recorded as m3. Relative water content (%) = (m1-m3)/(m2-m3)×100%.

### 2.8. Determination of ABA Contents

Fifty milligram of each leaf sample was ground to powder in liquid nitrogen. 500 µL extraction buffer (2:1:0.002 isopropanol/H_2_O/HCl, v/v/v) and 50 μL internal control (D2-ABA) were added and vortexed vigorously for 10 sec. The mixture was shaken at 900 rpm, 4°C, for 30 min; 1 mL chloroform was added; the mixture was vortexed and then shaken for another 30 min. After centrifugation at 8000 g, 4°C, for 10 min, the lower phase was transferred into a fresh clean tube and dried with nitrogen gas at room temperature. The sample was re-dissolved in 100 µL methanol and filtered through a 0.45-µm filter. Chromatographic separation was performed on a Waters Acquity UPLC I-Class (Waters Corporation) using a Poroshell EC-120 3-µm, 3.0 × 100-mm column at a flow rate of 300 µL min.^−132^

### 2.9. Data Analysis

All results in this study were performed in more than three replicates. SPSS 19.0 software (SPSS Inc., Chicago, IL, United States) was used for statistical analysis of experimental measurement data. Student’s t test was used to confirm the variability of results between treatments, respectively. *p* < .05 (*) and *p* < .01 (**).

## 3. Results

### 3.1. *Bioinformatics Analysis of* ZmPl1

**Figure 1.**
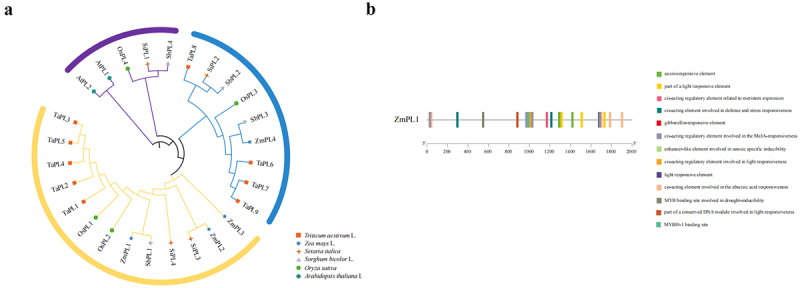
Bioinformatics analysis of *ZmPL1*. (a) Phylogenetic analysis of Papain-like cysteine proteases (plcp)-encoding enzymes from Ta (*Triticum aestivum* L.), zm (*Zea mays* L.), Si (*Setaria italica*), Sb (*Sorghum bicolor* L.), Os (*Oryza sativa*) and at (*Arabidopsis thaliana*). A neighbor-joining tree was generated using MEGAX with 1000 bootstrap replicates. (b) Analysis of promoter elements of *ZmPL1*.

Through a genome-wide search, 4, 9, 4, 4, 4, and 2 PL genes were identified in *Zea mays*, *Triticum aestivum*, *Setaria italica*, *Sorghum bicolor*, *Oryza sativa*, and *Arabidopsis thaliana*, respectively. Among these, the maize *ZmPL1* gene showed the highest homology with the *SbPL1* gene in *Sorghum bicolor* (Figure 1(a)). We analyzed the cis-acting elements of the *ZmPL1* promoter and identified multiple elements associated with stress responses, including drought-responsive elements (Figure 1(b)).

### 3.2. Analysis of ZmPl1 Gene Expression Pattern

**Figure 2.**
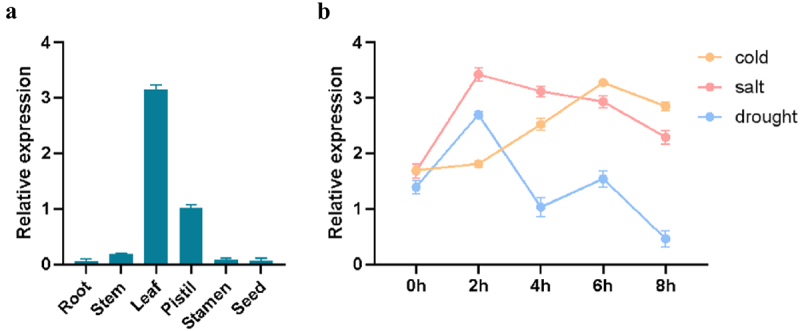
Analysis of *ZmPL1* gene expression patterns. (a) The expression of *ZmPL1* gene in different parts. (b) The expression of *ZmPL1* gene under abiotic stress (cold, salt and drought).

The expression of *ZmPL1* gene in different tissues was analyzed, and the results showed that *ZmPL1* gene was mainly expressed in leaves (Figure 2(a)). At the same time, the expression patterns of *ZmPL1* under cold stress, salt stress and drought stress were analyzed respectively. The results showed that the expression trend of *ZmPL1* gene under cold stress (4 ℃) gradually increased from 0 to 6 h and reached the maximum at 6 h, and then decreased at 8 h. The trend of gene expression under salt stress was that the expression level continued to increase from 0 to 6 h, and reached the maximum at 6 h, and then gradually decreased. Under drought stress, the trend of gene expression increased rapidly at 0–2 h, reached the maximum at 2 h, then gradually decreased and reached the minimum at 8 h (Figure 2(b)). The above results indicated that there were differences in the expression levels of *ZmPL1* gene under different stress conditions, among which the difference was more obvious under drought stress treatment, so it was inferred that *ZmPL1* gene was greatly affected by drought stress.

### 3.3. Subcellular Localization of ZmPL Protein

**Figure 3.**
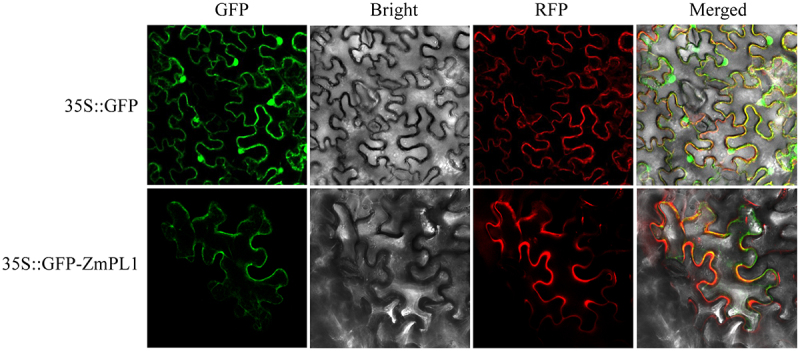
Subcellular localization analysis of ZmPL1 proteins in tobacco cells.

The fusion protein 35S:GFP-ZmPL1 was constructed by recombining the *ZmPL1* gene into the pCAMBIA1302 vector. Both the fusion protein and the control plasmid (35S:GFP) were separately transformed into *agrobacterium tumefaciens*. Subsequently, transient transformation was performed in tobacco leaf tissues, and the results were observed. The results showed that ZmPL1 protein was localized on the cell membrane (Figure 3).

### 3.4. *Functional Studies in* Saccharomyces cerevisiae

**Figure 4.**
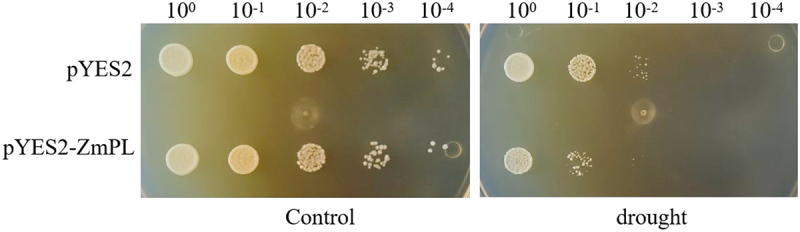
Heterologous expression of *ZmPL1* in yeast cells. Yeast cells were grown in control medium and medium containing 1.2 M mannitol.

We first investigated the drought tolerance function of *ZmPL1* in saccharomyces cerevisiae cells. In this study, recombinant plasmid pYES2-ZmPL1 and empty vector pYES2 were transferred into saccharomyces cerevisiae INVSc1, respectively. Drought stress was applied on SC-Ura+Galactose basal medium, and yeast cells with different concentration gradients were cultured. The results showed that on the medium containing 1.2 M mannitol, the yeast transferred with pYES2-ZmPL1 was significantly weaker than the yeast transferred with the pYES2 empty vector (Figure 4). The above results indicate that the *ZmPL1* gene was sensitive to drought stress.

### 3.5. Phenotypes and Growth Indicators of Transgenic Maize Plants During Germination Under Drought Stress

**Figure 5.**
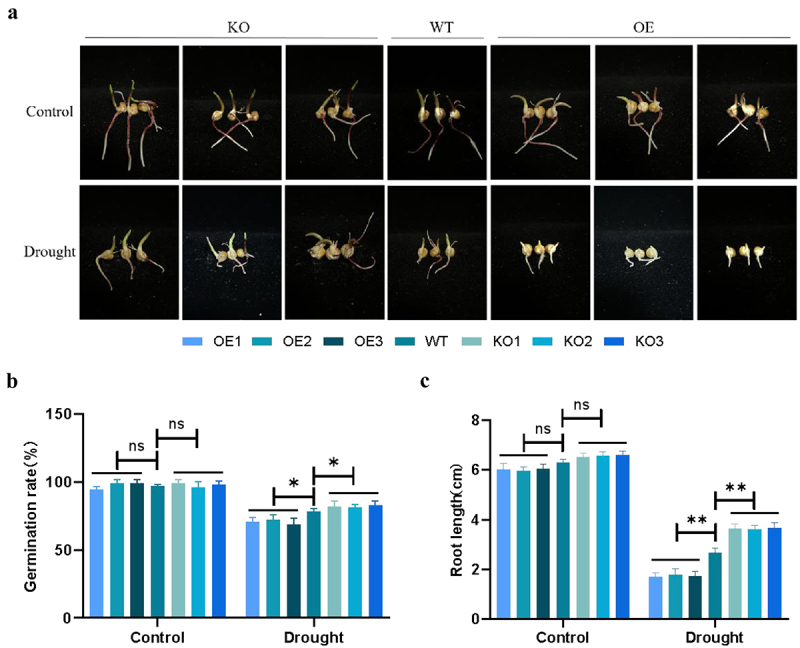
Knockdown of ZmPL alleviate the inhibition of seed germination rate and root length under drought stress.(a) seeds of WT and transgenic lines germinated on filter paper soaked of PEG6000. Scale bar = 0.5 cm. (B, C) statistics of germination rate and root length for different genotypes. Using Student’s t-test, asterisks indicate statistically significant differences (*p < .05; **p < .01). Data are shown as mean ± SD from three independent experiments.

In order to clarify the drought tolerance function of *ZmPL1* gene, further functional verification was carried out in maize. Firstly, the germination of different lines under drought stress was analyzed. It was found that the germination rate and root length of overexpressed maize were significantly inhibited compared with that of wild type (WT). On the contrary, the germination rate and root length of gene-edited plants were slightly inhibited compared with that of WT (Figure 5(a–c)). Therefore, it was speculated that overexpressed plants were more sensitive to drought stress, whereas gene-edited plants exhibited better drought tolerance.

### 3.6. Effects of Drought Stress on Transgenic Plants at the Seedling Stage

**Figure 6.**
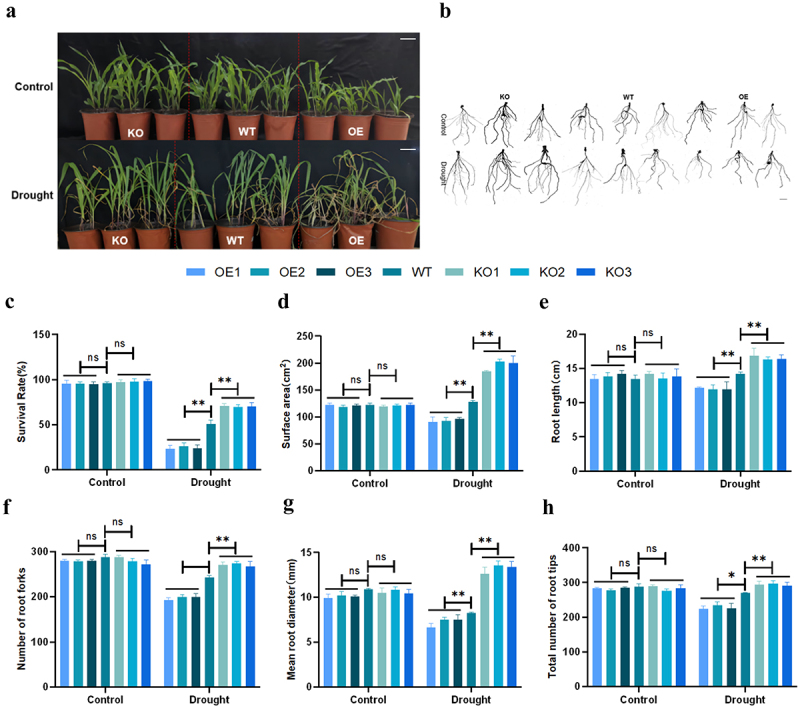
The overexpression of *ZmPL* reduced the survival rate of maize under drought stress. (a) Phenotype of WT and transgenic lines after drought treatment. Scale bar = 5 cm. (b) Root scanning results of WT and transgenic plants. Scale bar = 2 cm. (c-h) statistics of survival rate, surface area, root length, number of root forks, mean root diameter and total number of root tips for different genotypes. Using Student’s t-test, asterisks indicate statistically significant differences (*p < .05; **p < .01). Data are shown as mean ± SD from three independent experiments.

Next, we analyzed the phenotype of transgenic plants at seedling stage under drought stress treatment, and found that after drought stress treatment, overexpressed plants wilted more seriously than wild type plants, while gene-edited plants wilted less and had a higher survival rate. We also scanned the roots of WT and transgenic plants (Figure 6(a,c)). It was found that under drought stress, the roots of overexpressing plants were shorter than those of WT plants, and there was no significant difference in the number of root branches. But the gene-edited plants had longer roots, more root branches, and other root indicators were better than those of the WT and overexpression lines (Figure 6(b,d–h)). The above results showed that overexpression of *ZmPL1* increased the sensitivity of plant to drought, while gene editing of the *ZmPL1* gene could promote root development and increase the number of root branches, thereby enhancing the drought tolerance of maize.

### 3.7. Detection of Physiological and Biochemical Parameters of Transgenic Plants Under Drought Stress

**Figure 7.**
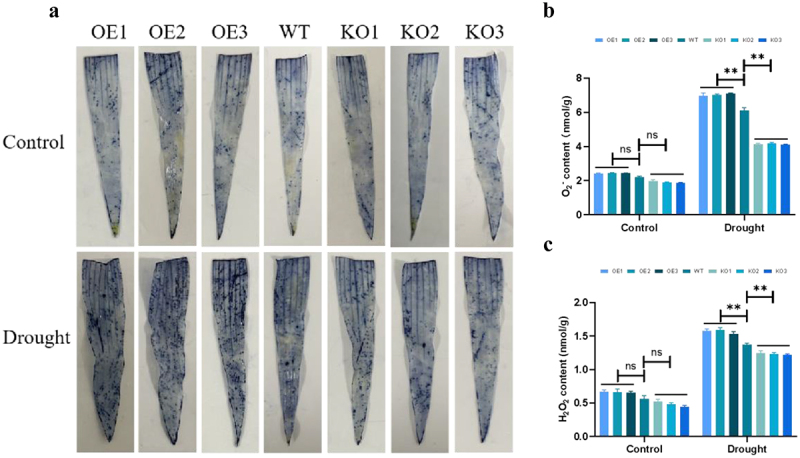
*ZmPL1* promoted the accumulation of reactive oxygen species (ROS). (a) Nitro-blue tetrazolium chloride (NBT) staining. Maize leaves were stained with NBT after drought treatment. (b, c) accumulation of O_2_^−^ and H_2_O_2_ in leaves of different lines. Using Student’s t-test, asterisks indicate statistically significant differences (**p < .01). Data are shown as mean ± SD from three independent experiments.

ROS plays a role in secondary metabolism in plants. Drought stress can lead to the imbalance of ROS, and the high levels of ROS can damage cell structures, affect the normal growth and development of plants, and even endanger the life of the plants.^9^ Therefore, the accumulation of ROS was detected in this study. Through NBT staining, it was found that under drought stress, compared with WT plants, the blue area of overexpression maize plants was larger, while the blue area of gene-edited plants was relatively smaller (Figure 7(a)). At the same time, a quantitative analysis of H_2_O_2_ and O_2_^−^ was conducted, and the study found that the contents of H_2_O_2_ and O_2_^−^ in overexpressed maize plants were higher, while the levels in gene-edited plants were relatively low (Figure 7(b,c)). The above results indicated that overexpression of *ZmPL1* promoted the accumulation of ROS.

**Figure 8.**
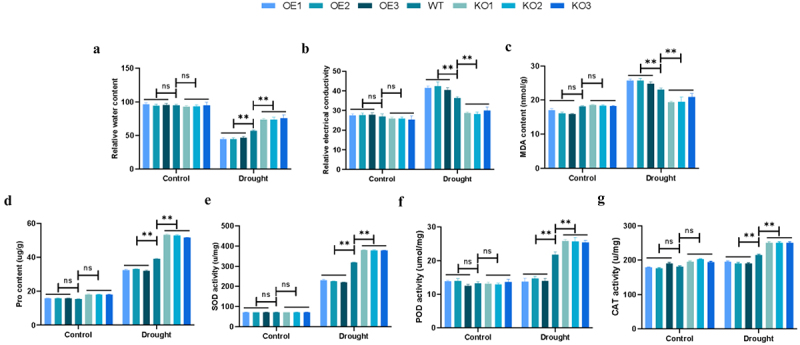
The physiological and biochemical indexes of transgenic lines were detected under drought stress. (a) Relative water content. (b) Relative electrical conductivity. (c) Malondialdehyde(MDA) content. (d–g) analysis of superoxide dismutase (SOD), catalase (CAT), peroxidase (POD) and ascorbic acid peroxidase (APX) activity. Using Student’s t-test, asterisks indicate statistically significant differences (**p < .01). Data are shown as mean ± SD from three independent experiments.

At the same time, other physiological and biochemical indicators were tested, and the results showed that under normal conditions, there were no significant differences in these indicators between WT and transgenic plants. However, under drought stress, the relative water content of the overexpressed plants was significantly lower than that of the WT plants, only 0.725 times that of the WT plants. The relative water content of gene-edited plants was significantly higher than that of WT plants, which was 1.19 times that of WT plants (Figure 8(a)). Moreover, compared with WT plants, the relative electrical conductivity and MDA content in the overexpression plants increased significantly, whereas the increases in the gene-edited plants were relatively slower (Figure 8(b,c)). The proline (Pro) content of the overexpressed plants was significantly lower than that of the WT plants, whereas the Pro content of the gene-edited plants was 1.62 times higher than that of the WT plants (Figure 8(d)). At the same time, we also measured the activity of antioxidant enzymes. Compared with WT plants, the activities of SOD, POD and CAT in overexpressed plants were significantly lower, only 0.76 times, 0.62 times and 0.90 times of the enzyme activities of WT plants, while the enzyme activities in gene-edited plants was significantly higher, with enzyme activities 1.06 times, 1.15 times and 1.15 times that of WT plants (Figure 8(e–g)). It was concluded that a large amount of ROS accumulated in the overexpression plants could not be removed, thus destroys the ROS homeostasis and enhancing the sensitivity of plants to drought. Knocking out *ZmPL1* was beneficial to maintaining ROS homeostasis and improving drought tolerance.

### 3.8. Effects of Transgenic Plants on the Expression of ROS Scavenging-Related Genes and Stress Response -Related Genes

**Figure 9.**
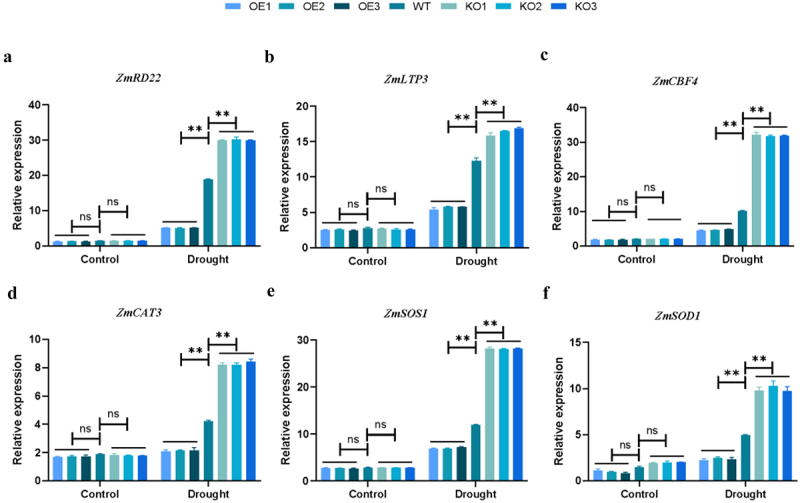
Expression levels of related genes in WT and transgenic plants under normal condition and drought treatment. (a – c) expression analysis of stress related genes. (d–f) expression analysis of reactive oxygen species (ROS) related genes.Genes.The expression level was normalized to that of maize *ZmACTIN1*. Using Student’s t-test, asterisks indicate statistically significant differences (**p < .01). Data are shown as mean ± SD from three independent experiments.

In order to further understand the regulatory mechanism of *ZmPL1* on drought stress, we analyzed the expression of known stress-related genes. Under normal conditions, the expression levels of *ZmLTP3*, *ZmRD22* and *ZmCBF4* genes in transgenic plants were not significantly different than those in WT plants. However, under drought stress conditions, the expression levels of these gene in the overexpression plants were significantly lower than those in the WT plants, while the expression levels of each gene in the gene-edited plants were significantly increased (Figure 9(a–c)). At the same time, we analyzed the expression of several genes involved in the ROS scavenging mechanism (*ZmCAT3*, *ZmSOS1* and *ZmSOD1*) and found that the expression of genes related to stress response had the same trend (Fig. 9d–f). These results indicated that under drought stress, overexpression of *ZmPL1* gene negatively regulated the expression of both stress-related and ROS scavenging genes, resulting in excessive accumulation of ROS in maize, making the plants more sensitive to drought stress, while knocking out these gene could enhance the drought tolerance of the plants.

### 3.9. ZmPl1 Responded to Exogenous Abscisic Acid

**Figure 10.**
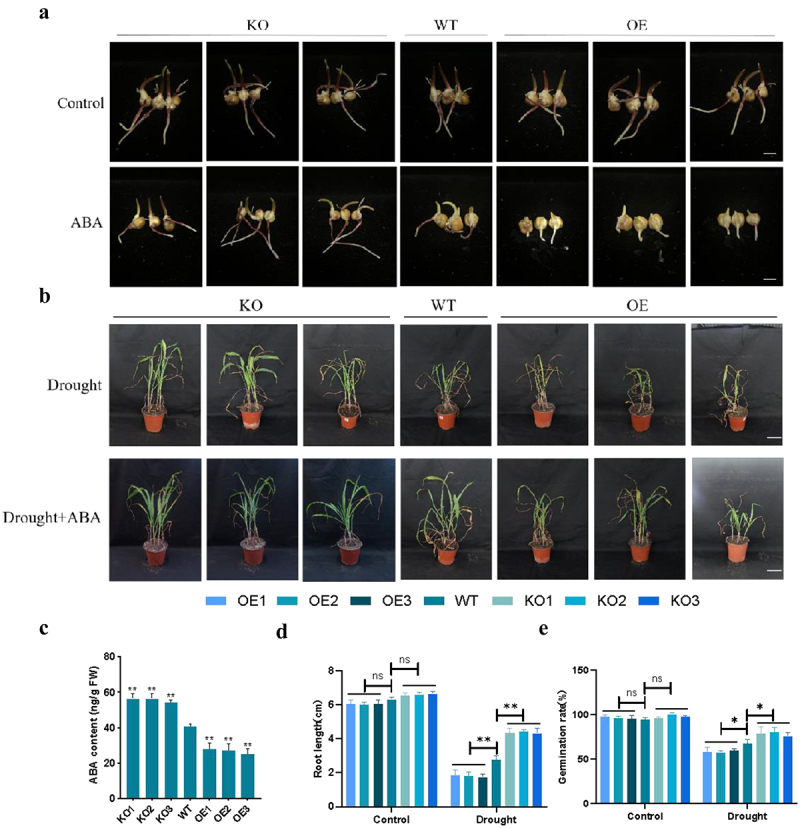
*ZmPL1* response to exogenous abscisic acid. (a) Seeds of WT and transgenic lines germinated on filter paper soaked of ABA; scale bar = 1 cm. (b) Phenotypic changes of transgenic lines and wild-type lines with or without ABA under drought stress. Scale bar = 5 cm. (c) ABA content of transgenic lines and WT seedlings. (d, e) statistics of root length and germination rate for different genotypes.

ABA affects the germination of plants and plays crucial roles in plant response to drought. The analysis of the *ZmPL1* promoter sequence showed that it contained ABREs (Figure 1(b)). We next measured ABA content. After drought treatment, *ZmPL1* overexpressing seedlings exhibited significantly lower ABA content compared to WT seedlings, whereas the gene-edited plants accumulated higher ABA levels than the WT seedlings (Figure 10(c)). We investigated the effect of ABA on seed germination. After 7 days of ABA treatment, the root length and germination rate of *ZmPL1* gene-edited plants was higher than that of the overexpression plants, indicating lower sensitivity to ABA (Figure 10(a,d,e)).

At the same time, we applied ABA treatment to each lines under natural drought stress at the seedling stage, observed the phenotype of transgenic lines and WT seedlings. Under drought conditions, the growth of *ZmPL1* overexpressing plants was significantly inhibited. However, after exogenous ABA treatment, the drought hypersensitive phenotype of *ZmPL1* overexpressing plants was restored (Figure 10(b)). These results suggested that the expression of *ZmPL1* reduced drought-induced ABA accumulation in seedlings, resulting in a drought-sensitive phenotype in seedlings.

## 4. Discussion

With the continuous change of global climate, the adverse effects of drought stress are increasingly intensified, so it is urgent to study new germplasm of drought-resistant maize through genetic engineering technology.^33^ Currently, the research on the function of Phylloplanin-like genes is mainly concentrated in tobacco, and the research on the function of Phylloplanin-like genes in other species is at a blank stage. In tobacco, the T-Phylloplanin-like protein had been demonstrated to enhance plant resistance to abiotic stresses, including drought, salinity, and cold.^20^ By analyzing the promoter sequence of *ZmPL1*, we found that it contains several elements related to stress (Figure 1(b)). This study had subsequently analyzed the expression of this gene under different stress treatments. The qRT-PCR results showed that *ZmPL1* responded to drought, salt and cold, and was significantly affected by drought stress (Figure 2(b)), which was consistent with the results of previous studies.^21^ Subcellular localization results showed that *ZmPL1* mainly existed on the cell membrane (Figure 3). *Saccharomyces cerevisiae* cells, a single-celled eukaryote, offer advantages such as rapid growth, high precision, and the ability to efficiently express eukaryotic proteins, making it an ideal model organism for studying gene function related to stress tolerance.^34^ For example, in soybean, overexpression of genes such as *GsTIP2–1*, G*sTIP2–2*, *GsINT1*, *GsSUC4*, and *GsACA11* had been shown to enhance the salt-alkali stress tolerance in Saccharomyces cerevisiae cells.^35^ Overexpression of *ZmDHN15* had been shown to improve the tolerance of yeast transformants.^36^ In this study, the *Saccharomyces cerevisiae* expression system was used to investigate the stress tolerance function of *ZmPL1*. The results showed that *ZmPL1* transgenic yeast was highly sensitive to drought stress (Figure 4).

When plants are exposed to drought stress, their external morphology and internal secretions will change.^37^ When plants are exposed to drought stress for a long time, they will respond to the stress by adjusting their root morphology and increasing their root length.^38^ In this study, we observed the phenotypes of each lines at the germination and seedling stages under drought stress and found that the germination and seedling survival rates of the *ZmPL1* knockout lines were significantly better than those of the overexpression lines and the WT lines (Figures 5(a,b) and 6(a)). At the same time, the root length, number of lateral roots and other root indicators of the knockout lines were also better than those of the overexpression lines (Figures 5(c) and 6(b,d–h)). Therefore, the root morphology was regulated by knocking out the *ZmPL1* gene, which improved the adaptability to drought stress. Relative electrical conductivity, MDA and Pro content are important indicators for evaluating osmotic pressure damage to plant cell membranes.^11,39^ In maize, the MDA content of *SAMDC* overexpressing plants was significantly reduced, and the expression of *CBF* and cold-responsive genes was significantly increased. Overexpression of the *SAMDC* gene effectively improved the cold tolerance of maize.^40^ In wheat, after drought treatment, the membrane leakage rate in the leaves of the *TaSNAC11-4B* overexpression lines was significantly higher than that of Col-0, indicating that the expression of *TaSNAC11-4B* was more sensitive to drought stress.^41^ Based on the above research results, this study also measured the relative electrical conductivity, MDA and Pro content. The results showed that under drought conditions, the relative contents of MDA and Relative electrical conductivity of the overexpression plants were higher than those of the gene-edited plants, while Pro content was lower than that of the gene-edited plants. These results indicated that overexpression of *ZmPL1* increased the sensitivity of plants to drought (Figure 8(a,b,c)).

Under adverse environmental conditions, plants experience an increase in reactive oxygen species (ROS) content. Excessive accumulation of ROS can damage DNA, RNA, proteins, and membranes. Therefore, plants will produce more antioxidant enzymes to remove the accumulation of ROS and maintain ROS balance and intracellular redox homeostasis.^42,43^ During this process, the expression and regulation of related genes in plants are also altered. By measuring the expression levels of stress-related genes and ROS scavenging-related genes in plants under abiotic stress, the drought tolerance of plants can be more accurately reflected.^44–46^ Under drought stress, overexpression of the *GmDHN9* gene in *Arabidopsis thaliana* resulted in higher germination rate, longer root length, increased chlorophyll content, proline accumulation, relative water content, and antioxidant enzyme activity compared to WT plants. Additionally, lower levels of O_2_^−^, H_2_O_2_, and MDA content were observed in the overexpressing plants. These findings suggested that the *GmDHN9* gene could regulate the homeostasis of ROS and enhance plant drought tolerance.^47^ Low expression levels of *OsSPL10* and its downstream gene *OsNAC2* could reduce the expression of *OsAP37* and increase the expression of *OsCOX11*, thereby preventing the accumulation of ROS and programmed cell death (PCD).^48^ Under abiotic stress, *IbBBX24* and *IbPRX17* overexpressing lines exhibited higher peroxidase activity and lower H_2_O_2_ accumulation compared with the WT. RNA sequencing analysis revealed that *IbBBX24* regulated the expression of genes encoding ROS-scavenging enzymes, including PRXs.^49^ In this study, we detected the accumulation of ROS, the activities of corresponding antioxidant enzyme and the expression of related genes. Compared with the overexpression and WT plants, the gene-edited plants showed lower levels of H_2_O_2_ and O_2_^−^, along with higher activities of SOD, POD, and CAT (Figures. 7(a–c), 8(e–g)). Meanwhile, the expression levels of stress-related genes (*ZmLTP3*, *ZmRD22* and *ZmCBF4*) and ROS scavenging-related genes (*ZmCAT3*, *ZmSOS1* and *ZmSOD1*) were higher in the gene-edited plants compared to the overexpression and WT plants (Figure 9(a–f)). This suggested that knocking out the *ZmPL1* gene reduced excessive ROS accumulation, maintained ROS balance, enhanced the plant’s drought stress tolerance, and might have improved maize yield. This study laid a theoretical foundation for the innovation of maize germplasm resources with high quality, high yield and strong stress resistance.

ABA is an important plant endogenous hormone involved in the regulation of plant development. In plants subjected to drought stress, plants reduce water loss by stomatal closure and increase ABA content to enhance drought tolerance.^50^ For example, *Zmhdz9* promoted drought stress tolerance in maize by modulating ABA.^51^ MAPK-like protein 1 positively regulated maize seedling drought sensitivity by suppressing ABA biosynthesis.^32^ *ZmASR1* knockout lines generated with the CRISPR – Cas9 system showed lower ROS accumulation, higher ABA content, and a higher degree of stomatal closure than wild-type plants, leading to higher drought tolerance.^52^ In this study, exogenous application of ABA alleviated drought stress injury in *ZmPL1* overexpressing plants. *ZmPL1* regulated plant tolerance to drought stress through the ABA signaling pathway (Figure 10).

## 5. Conclusions

This study investigated the function of *ZmPL1* in maize. The expression level of *ZmPL1* changed under drought stress. The detection of phenotypic and physiological and biochemical indicators showed that overexpression of the *ZmPL1* gene enhanced the sensitivity of maize to drought. *ZmPL1* regulated antioxidant system and ABA signaling pathway under drought stress. In conclusion, *ZmPL1* is an important gene related to drought tolerance, which has important development prospects in the breeding of drought-tolerant maize varieties in the future.

## Supplementary Material

Supplemental Material

## Data Availability

Data will be made available on request.
