# Supplementary material for: CRISPR-Cas9-mediated editing of ZmPL1 gene improves tolerance to drought stress in maize
Source: GM Crops Food. 2025 Jan 17;16(1):1–16. doi: 10.1080/21645698.2024.2448869 (PMC12758173; doi:10.1080/21645698.2024.2448869)
Supplement: Supplemental Material [file KGMC_A_2448869_SM5043.zip › Supplementary Figures-reviews clean.doc]

Fig. S1 Construction of pCAMBIA3301-ZmPL1 vector. (A) Structure diagram of pCAMBIA3301-ZmPL1 carrier. (B) PCR detection of *ZmPL1* gene plasmid; M: DL2000; P: Cloning vector plasmid; N: negative control; 1-3 Recombinant vector plasmid. (C) pCAMBIA 3301-ZmPL1 double digestion; M: DL2000; 1: recombinant vector plasmid. (D) Sequencing comparison results

Fig. S2 Construction of pCXB053-ZmPL1 vector. (A) Structure diagram of pCXB053-Phylloplanin-like vector. (B) pCXB053-ZmPL1 plasmid PCR detection；M: DL2000; 1-4 Recombinant vector plasmid. (C) Identification of pCXB053-ZmPL1

Fig. S3 Target detection results of gene editing plants in T_2_ generations. (A)Nucleotide sequences of gene-edited lines. Note: S stands for base substitution and - is base deletion. (B)Amino acid sequence of a gene-edited line.

Fig. S4 T_2_ overexpressed plants were detected by PCR. (A) PCR detection of T_2_ overexpressed plants. (B) 35S detection of T_2_ overexpressed plants. (C) NOS detection of T_2_ overexpressed plants. (D) PCR detection of T_2_ generation gene editing plants. (E) 35S test of T_2_ generation gene editing plants. (F) NOS detection of T_2_ generation gene editing plants; M: DL2000; P: recombinant vector plasmid; N: negative control; CK: non-transgenic plants; 1-3: Transgenic plants

Fig. S5 Expression analysis of transgenic lines (A) Expression analysis of *ZmSAG39* in overexpression of *ZmSAG39* lines. 1-3: Transgenic plant lines. (B) Expression analysis of *ZmSAG39* gene in gknock-out lines. 1-3: Transgenic plant lines. Student’s t-test was performed, and significant differences are indicated by: **P < 0.01. Data are shown as mean ± SD from three independent experiments.
