# Supplementary material for: CRISPR-Cas9-mediated editing of ZmPL1 gene improves tolerance to drought stress in maize
Source: GM Crops Food. 2025 Jan 17;16(1):1–16. doi: 10.1080/21645698.2024.2448869 (PMC12758173; doi:10.1080/21645698.2024.2448869)
Supplement: Supplemental Material [file KGMC_A_2448869_SM5043.zip › Supplementary Tables.doc]

Table S1. Primers uesd in this study

| Name | Primer sequence（5'-3'） |
| --- | --- |
| Q-ZmPL1-F | ACCGGGAACTCCATTGACC |
| Q-ZmPL1-R | CCTCGAAAGTTGCTTATGAACAG |
| Actin-F | TTGAGGTAGGATGAGACT |
| Actin-R | GGAGTGAAGCAGATGATT |
| ZmPL1-F | AGACGAAGCGGAGCTGATAA |
| ZmPL1-R | CGGCCGCTACACGTATTGA |
| 1302-ZmPL1-F | acgggggactcttgaccatggAGACGAAGCGGAGCTGATAA |
| 1302-ZmPL1-R | tctcctttactagtcagatctCGGCCGCTACACGTATTGA |
| 3301-ZmPL1-F | actcttgaccatggtAGATCTAGACGAAGCGGAGCTGATAA |
| 3301-ZmPL1-R | ggggaaattcgagctggtcaccCGGCCGCTACACGTATTGA |
| pYES2-ZmPL1-F | actatagggaatattaagcttAGACGAAGCGGAGCTGATAA |
| pYES2-ZmPL1-R | tgatggatatctgcagaattcCGGCCGCTACACGTATTGA |
| CRISPR-ZmPL1-F | TGTCGGCCTTGCCTTCCCTTCGG |
| CRISPR-ZmPL1-R | CCCTCGTGCAGCTCGTGTGCGGC |
| Bar-F | TCAAATCTCGGTGACGGGC |
| Bar-R | ATGAGCCCAGAACGACGC |
| 35-F | TAGAGGACCTAACAGAAC |
| 35-R | CCGTGTTCTCTCCAAATG |
| Nor-F | GAATCCTGTTGCCGGTCTTG |
| Nor-R | TTATCCTAGTTTGCGCGCTA |
| Q-ZmLTP3-F | GGCTTTCGCTTTGAGGTTCTTC |
| Q-ZmLTP3-R | ATGCTAACACCGCACTTTCCAG |
| Q-ZmRD22-F | GCG GGCGGGCGGCGGGCGCCT G |
| Q-ZmRD22-R | TCAGCCGCCGCGGGTCCAGACGACG |
| Q-ZmCBF4-F | CTGG GAGGAAGAAGTTTCGTGAG |
| Q-ZmCBF4-R | CGTTATGATT CCAGCCAACTTCC |
| Q-ZmCAT3-F | GCGTTGAAACCTAACCCGAAAA |
| Q-ZmCAT3-R | AAACCCTCCATGTGCCTGTAATCTT |
| Q-ZmSOD1-F | TGAGGGTGTTACGGGGACTATCT |
| Q-ZmSOD1-R | GTGAAGGTGGCAGTTCCATCATCTC |
| Q-ZmSOS1 -F | TCATCATCCTCACAATGGCTCTAA |
| Q-ZmSOS1-R | ACCAACTTGCGTGGGACAACTTTA |
